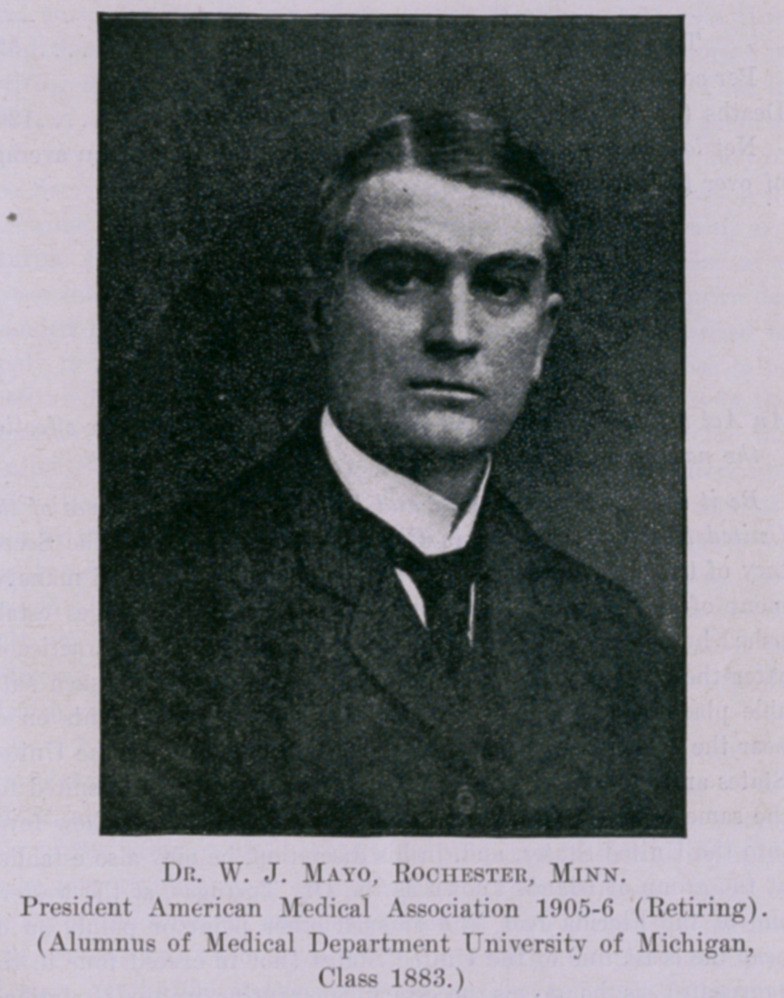# Bon-Bons

**Published:** 1906-07

**Authors:** 


### Bon-Bons.

Dr. T. H. Nott, of Goliad, an ex-president of the Texas State Medical Association, died at Goliad on June 10th, of heart disease.

It is one of the greatest tragedies of life that every truth has to struggle to acceptance against honest but mind-blind students. —*Osler.*

The Fellows Medicine Manufacturing Company, 26 Christopher street, New York, manufacturers of the famous Fellows Compound Syrup of Hypophosphites, is now "The Fellows Company of New York."

"Yes, colored physicians are eligible to membership in the American Medical Association, and there are at present colored members in that body."—*Dr. Geo. H. Simmons, Secretary A. M. A., and Editor Journal A. M. A., in reply to the "Red Back's" inquiry.*

Significant.—The physicians of Georgia are organizing an Independent State Medical Association. They will throw off the yoke of the Octopus. Success to them. Let the example be followed by every other Southern State, and let us have a Confederacy of Southern Medical Associations.

Editor "Red Back": The enclosed dollar pays for the dear old "Red Back" a year. Keep on sending it. I wish it could live and prosper "as long as water runs and grass grows."

William Osborne,
Bluffton, Texas.

The Independent Press should hold a convention and protest against the Medical Journal Trust. In union only is there strength. We can not effectively fight the combination in detail. But one thing is certain: the Czar is bound to go. He is marked for slaughter. A juster, more conservative, more popular and less presumptuous man should be put in his place.

THE retiring president, in his addresses at the Boston meeting, called attention to the wide-spread and growing dissatisfaction with the way the Association is run, and with the policy of the *Journal,* yet nothing was done. On the contrary, one or two turns of the screws were made, as pointed out elsewhere. The clique will do well to heed the mutterings of the storm that is surely coming. It will break at Atlantic City next June.

---

"LET the doctors dissect each other, d—n them!" —*Senator Davidson, Candidate for Lieutenant-Governor.*

---

"THE Fort Worth doctors are a most ungodly set of butchers." —*Senator Hanger.*

---

"IF JESUS CHRIST were crucified in Texas today, the doctors would steal his body before night."—*Senator Chambers.*

---

PRIMARIES: July 28th. "Remember the Main(e)" issue.

---

VOTE for Hon. F. F. Hill for Lieutenant-Governor, the friend of legitimate medicine and legislation.

---

SEASICKNESS: The "Girard Mixture" claimed to be a specific. —Col. A. C. Girard, Surgeon U. S. A., retired; late Assistant Surgeon-General U. S. A., San Francisco, Cal., read a paper at Lisbon (International Medical Congress, April 17, 1906), in which he claimed that 1/120 grain atropin sulphate and 1/60 grain strychnin sulphate injected into an adult "at the commencement of the voyage, or when the sea begins to be rough," will prevent seasickness. He adduces, in support of the claim, much testimony from naval surgeons and other medical men, as well as from passengers. This dose may be repeated at short intervals, if necessary. "Incipient dryness of the throat or disturbance of vision indicate that atropinism has been reached," when, the doctor says, "in every instance of my observation, the symptoms of seasickness disappeared" (or did not appear?).

---

RESOLUTION PASSED BY THE BRAZOS VALLEY MEDICAL ASSOCIATION, WEDNESDAY, MAY 30, 1906, AT HEARNE, TEXAS.

WHEREAS, Many of the life insurance companies of this country have recently by an apparent united action reduced their fees for examinations to a sum which, we believe, is below a reasonable remuneration for our services, and that we do not believe that retrenchment has reference to the medical department, which seems to have escaped any charges of graft or extravagance, be it

*Resolved*, That we believe this reduction is unjust and it is the sense of this Association that no member shall make an examination for an old line insurance company for less than five dollars ($5.00), and that we insist that this be paid by the company rather than any part shared by the agent. We also recommend that the members of this Association render no service to any company that refuses to pay this reasonable compensation.

Dr. W. A. Bedford of Franklin was elected President of the Brazos Valley Medical Association, Dr. R. E. Bledsoe, of Somerville, Vice-President, and Dr. A. S. Epperson, of Cameron, Secretary and Treasurer. Next meeting at Somerville in November next.

---

### CONGRATULATIONS AND APPRECIATION.

New York, June 16, 1906.

*Texas Medical Journal, F. E. Daniel, M. D., Editor, Austin, Texas.*

Dear Doctor: The June issue of your valued journal is at hand. The editorial make-up is, as usual, bright and interesting. There is no question as to your standing on any matter of right and wrong, and the influence of the "Red Back" is felt very widely. We wish to congratulate the "Red Back" upon the fact that it has attained its majority, and just at this time when the services of strength and character are so much needed in medical journal life, a celebration of this kind should be marked in a little gift. Knowing your propensity for Glyco-Thymoline, we are sending you, by express prepaid, a little package for your personal use.

Wishing you and your journal continued success, we remain,

Yours very truly,
Kress & Owen Company,
Samuel Owen,
President.

---

### ECHOES FROM BOSTON.

The American Medical Editors' Association held a very satisfactory annual meeting in Boston on Monday, June 4th, under the presidency of Dr. Henry Waldo Coe, of Portland, Oregon. Officers for the ensuing year were elected as follows: President, Dr. James Evelyn Pilcher, of Carlisle, Pa.; vice-presidents, Dr.

Frank P. Foster, of New York, and Dr. Charles F. Taylor, of Philadelphia; secretary and treasurer, Dr. Joseph McDonald, Jr., of New York. The success of the annual banquet, at the University Club, was largely due to the endeavors of Dr. Charles Greene Cumston, of Boston.

At that meeting Dr. W. C. Abbott, of Chicago, the editor and publisher of the *Journal of Clinical Medicine,* spoke as follows in defense of advertising proprietary medicines to physicians:

"It has become the fashion in these strenuous days to say uncomplimentary things about the advertiser. No doubt he deserves some of the reproaches which are being heaped upon him, for he must make good. Is this not true also of the religious leader, the social reformer, and the scientist? To be sure it is. * * *

"There is not a particle of doubt that there are many excellent remedies all about us which have been neglected or never discovered because no one has ever taken time or spent money to investigate them thoroughly. This means advertising.

"How far will the one-sided, hysterical critic go along these lines? How much is he himself doing without hope of reward? What does a physician or surgeon do who has discovered a new bacterium, a new method of operating, no better perhaps, nor no worse than others? He advertises it. * * *

"It would be unfair to the manufacturers to demand of them qualitative and quantitative statements regarding every ingredient, together with detailed method of manufacure. What incentive would there be for any one to build laboratories, to spend time, labor and money, if, after discovering something valuable, he was compelled to disclose it, according to the provisions of the proposed legislation, to have the product at once pirated and imitated by persons who have nothing at stake and no financial interests and who could afford to sell the product at lower prices than the originator?

"The physician must know what he is giving the patient, but he does not care for unimportant details which give the product individuality.

"Must the formula be printed with the advertising each and every time? Not at all. This absurd demand could have originated only in the brain of an overzealous, perhaps crack-brained reformer. Let the honest advertiser, be he proprietary man or otherwise, stand for his rights, as he surely will! Let the fair, square journal support them all as it should, and as we will (cheers), and out of all this should come good for the worthy advertiser, and condemnation and disaster for the fraud and fakir, and a final solution on the right lines of many of the problems which now perplex us."—*Journal A. M. A.*

A Proposed Investigation of the Association's Affairs.—Dr. H. O. Walker, of Detroit, offered the following resolution:

"Mr. Chairman: Since the address of our worthy president of yesterday indicated the sentiment of uncertainty and distrust relating to the management of our *Journal* and sundry other matters, we naturally feel that these criticisms are both unjust and unfair, and yet every effort should be made to dissipate this feeling, at least from the minds of the members of this association.

"Therefore, Mr. Chairman, I beg leave to offer the following preamble and resolutions:

"Whereas, The membership of the American Medical Association, numbering 19,285, is scattered throughout all the States and Territories; and

"Whereas, The affairs of the association are so intricate that it is difficult to make them clear to all; and because of these facts, there has arisen the sentiment which bids fair to become disagreeably large, unless the causes upon which it feeds be removed, viz., ignorance of the real truth; therefore be it

"*Resolved,* That a committee of five, namely, G. Frank Lydston, of Chicago; Frederick Holme Wiggin, of New York; A. H. Cordier, of Kansas City, Mo.; Duncan S. Eve, of Nashville, Tenn., and D. W. Graham, of Chicago, be appointed by the House of Delegates of the American Medical Association and instructed: First, to make an exhaustive study of the affairs of the association, its *Journal,* etc. Second, to employ an auditing expert to go over all the books of the association; to have power to summon officers and employes of the association before it; to give needful testimony; and in such other ways as it may deem best to secure all facts necessary for such independent report as may be needful to accomplish its purpose.

"*Resolved,* That a sum of money be appropriated sufficient to defray the actual expenses of this study.

"*Resolved,* That this committee report to the House of Delegates at their next meeting, in 1907."

On motion, the resolutions were laid on the table.—*N. Y. Med. Journal.*

I learn that Dr. Philip M. Jones, editor of the *California State Journal,* moved to reconsider the vote to lay this resolution on the table. Dr. Jones' motion was *lost!* The *New York Medical Record* (June 16), speaking of the refusal to permit an investigation of the Association's finances, says:

"This meeting had promised to be politically more interesting than some of its recent predecessors, for it was rumored that a strenuous effort would be made to dismiss the secretary-editor. So great has become the dissatisfaction with his administration that this endeavor might have been successful had the general body any voice in the management of the Association, but as the society is ruled by the House of Delegates, whose members are appointed before the meeting each year by the State societies and not by the Association itself, the change of dynasty was not effected. The feeling of opposition among the members was indeed so strong that the retiring president deemed it wise to speak of it, to assure the delegates that everything was as it should be, and to avow that he personally knew nothing of the existence of any inner ring or clique. That assurance was, of course, unnecessary, for if there were an inner circle of 'bosses' such an ephemeral officer as the president would not be likely to be admitted to its councils. Another evidence of the general dissatisfaction with the management of the Association was the resolution presented in the House of Delegates calling for the appointment of a committee to look into the affairs of the treasurer and secretary. Drs. Billings and Simmons must now regret the shortsightedness of their friends in tabling this resolution, for they will have to be investigated some day and the sooner the disagreeable job is over the better. As Dr. Walker, of Detroit, stated in introducing his resolution, the criticism of the management has been so bitter because the majority of the members do not understand the intricacies of the business of the Association; the object of the proposed investigation was, therefore, to clear the officers of unjust suspicion and to demonstrate to all that they had acted solely in the interests of the Association, as they understood them, uninfluenced by motives of self-seeking. The unwise tabling of these resolutions will defer what the unhappy officers might resent as the indignity of an investigation, but it will, on the other hand, strengthen the suspicion in the minds of many that certain things may have been done which would not bear the light of an investigating committee's report. So far from quieting suspicion this evading the issue will be almost certain to increase it. As the preamble to Dr. Walker's resolution read: 'because of these facts, there has arisen the sentiment which bids fair to become disagreeably large, unless the cause upon which it feeds be removed, viz., ignorance of the real truth,' but the only way of making the real truth known has been stopped by the friends of the accused. These two officials are now in the unenviable position of serving a body, a large minority, if not the majority, of the members of which look upon them with distrust, yet their foolish friends refuse to permit an investigation which they must surely know is the only means of restoring confidence in their integrity. The opposition to an investigation could not have been on account of the constitution of the committee named in the resolution, for the Association could trust such men as Lydston and Graham, of Chicago, Wiggin, of New York, Cordier, of Kansas City, and Eve, of Nashville, to do their work conscientiously and make a report on the facts as they found them, free from any taint of prejudice or favoritism. For the sake of the Association, as well as of the officers mentioned, it is a great pity this well-intentioned endeavor to get at the truth and make it known was suppressed."

Report of the Chairman of the Board of Trustees.—Dr. T. J. Happell, of Tennessee, the chairman of the board of trustees, announced the net assets of the Association is nearly $238,000. The income from all sources for the past year had been about $275,000. The total expenses for the year had been approximately $250,000, leaving a net annual income of about $25,000, in round numbers.—*N. Y. Med. Journal.*

The Secretary's Report.—Dr. George H. Simmons read his report as secretary of the Association, in which he announced that there had been a most satisfactory gain in the number of the membership. During the year 4351 new members had been enrolled, making the total membership 23,636. The prospects for further gains of membership in the immediate future were most encouraging, as the work of organization of the profession was proceeding very satisfactorily.—*N. Y. Med. Journal.*

Amendments to the Constitution and By-Laws.—One amendment, proposed two years ago and adopted last year, was modified so as to make it possible for the board of trustees, by a unanimous vote, to change the place of meeting determined upon by the House of Delegates if it seemed advisable because satisfactory arrangements for transportation or accommodation could not be secured, provided the change was made at least four months before the time selected for the meeting.—*Journal A. M. A.*

THE ATTENDANCE AT BOSTON was unprecedented in numbers. There were registered "about 4700." This, of a membership of 23,636 (see report), is about 19 per cent, and of the physicians of America (about 120,000), it is less than 4 per cent!

STRICT RULES were enforced to admit none but members wearing badges. Outsiders, however numerous and respectable, were not admitted to the scientific body or sections, and, of course, not to House of Delegates and festivities. See official reports. Another method of driving them in.

DR. JOSEPH D. BRYANT, NEW YORK,

President-elect American Medical Association, 1906-7.

Dr. Bryant is Professor of the Principles and Practice of Surgery, Operative and Clinical Surgery in the University and Bellevue Medical College; Consulting Surgeon to the Hospital for Ruptured and Crippled; Visiting Surgeon to Bellevue and St. Vincent Hospitals; author of Bryant's System of Surgery, etc.

DR. G. B. FOSCUE, WACO, TEXAS.

President Texas State Medical Association.

Dr. Foscue was born near Jefferson, Texas, June 21, 1860. He received his literary education at the Kellyville Academy. Graduated in medicine at Long Island College Hospital, Brooklyn, N. Y. 1883; located at Callisburg, Cooke county, Texas, same year, and moved to Waco in July, 1884, where he has resided since. Was married to Miss Sallie E. Rowell, of Jefferson, in 1886. Is a member of several local and district societies and of the State and National Medical Associations since beginning the practice of medicine. He has done much post graduate work and has held several positions of honor in the various medical societies. During the Spanish war Dr. Foscue was appointed by the Surgeon General of the United States army to serve on a board to examine medical officers for the army. Elected councilor for the Twelfth District at the Austin meeting of the State Association in April, 1904, and President without opposition at Fort Worth meeting in April, 1906.

DR. W. J. MAYO, ROCHESTER, MINN.

President American Medical Association 1905-6 (Retiring).

(Alumnus of Medical Department University of Michigan, Class 1883.)

"Society appreciates the saving of a sick person's life by the skilled physician, but fails to see the priceless gifts to the human race made by preventive medicine and sanitary science. It views everything in detail, and misses the perspective. We have failed to secure the support of the mass of the people to much needed sanitary reforms, because we have appealed to them as one individual to another, without the weight of an authoritative organization." Presidential address, A. M. A., Boston.

---

STATUS OF THE MEMBERSHIP A. M. A. Recapitulation:

| | |
|---|---|
| New members enrolled during the year | 4351 |
| Less 3756 New York new code men come back (a thing that can not occur again | 3756 |
| Total gains | 595 |
| Per contra: | |
| Deaths (171), resignations and dropped | 1208 |

Net *loss* (excepting the new code increment), 613, or an average of over fifty each month.

## Figures and Tables

**Figure f1:**
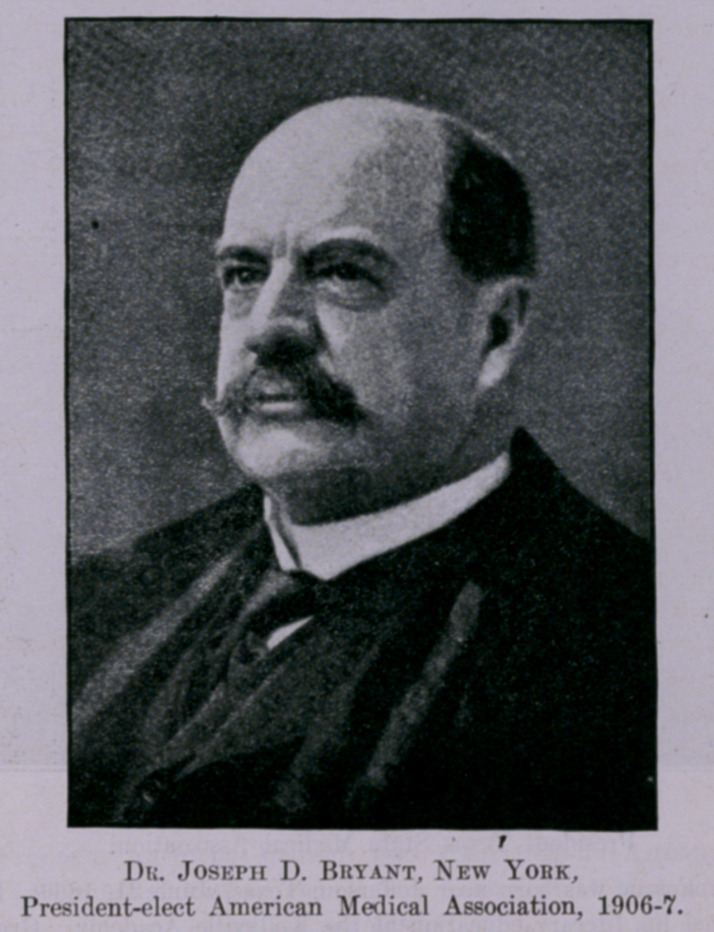


**Figure f2:**
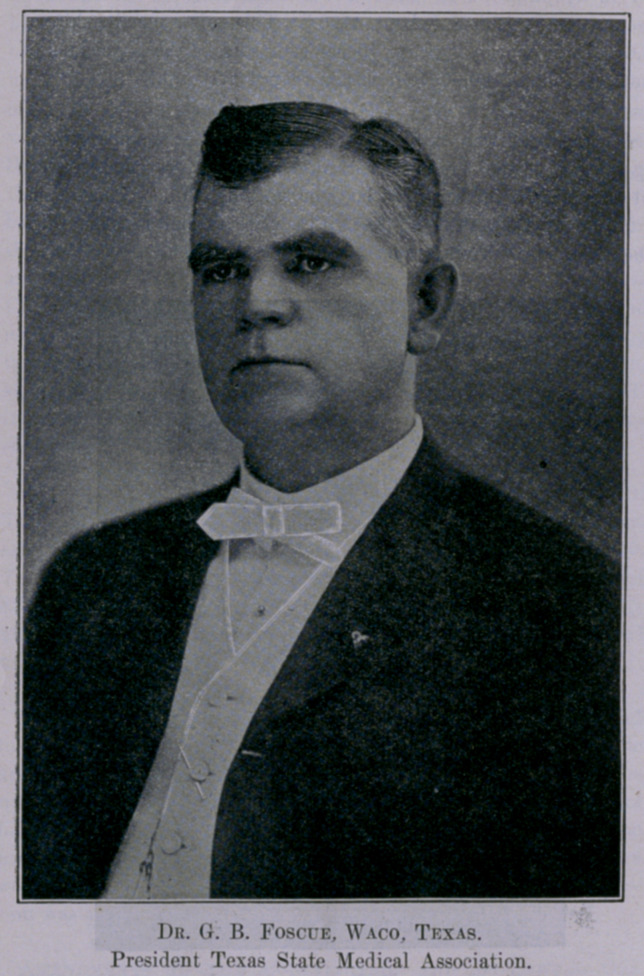


**Figure f3:**